# Supplementary figures and images for: Blood pressure and heart rate variability responses following an acute bout of vinyasa yoga and a prolonged seated control: A randomized crossover trial
Source: PLoS One. 2023 Nov 28;18(11):e0294945. doi: 10.1371/journal.pone.0294945 (PMC10684087; doi:10.1371/journal.pone.0294945)

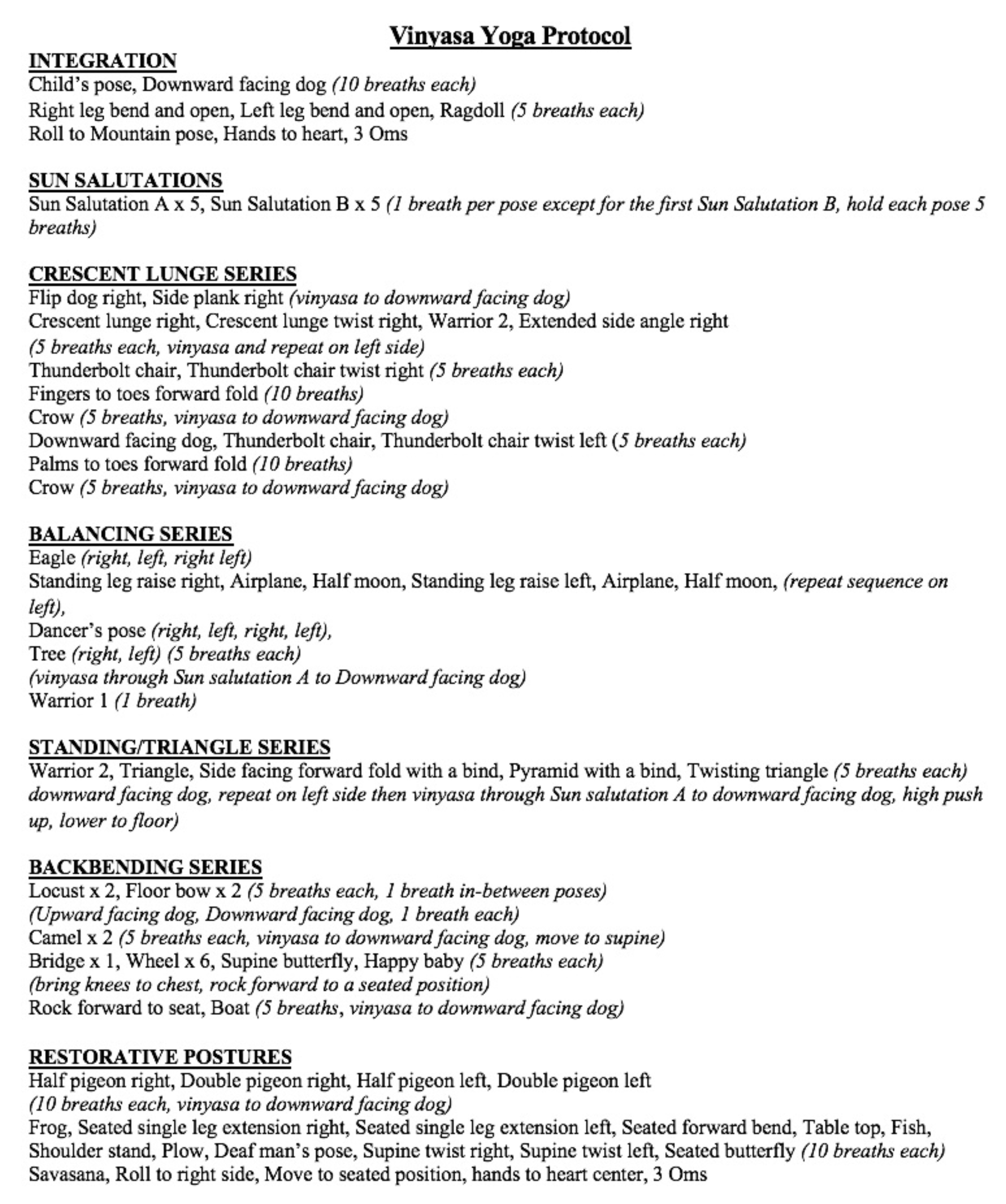

Supplement: S1 Fig — Poses are listed in order of how they were performed, and the number of breaths in each pose is listed after the pose name. (TIFF) [file pone.0294945.s001.tiff]
